# Editorial: Insights in aging, metabolism and redox biology

**DOI:** 10.3389/fragi.2024.1432858

**Published:** 2024-07-01

**Authors:** Changhan Lee, Jianhua Zhang

**Affiliations:** ^1^ Leonard Davis School of Gerontology, University of Southern California, Los Angeles, CA, United States; ^2^ USC Norris Comprehensive Cancer Center, Los Angeles, CA, United States; ^3^ Department of Pathology, University of Alabama-Birmingham, Birmingham, AL, United States

**Keywords:** aging, Nrf2, lifespan, BAG-5, PU.1, O-GlcNAc, STIM1, dauer

## Contribution to the field

In this Insights in Aging, Metabolism and Redox Biology Research Topic, we summarized the cutting-edge aging research that provide insights into where we are and where we want to be in Aging research with 9 articles.

Zhang discusses the significant intersections between aging, metabolism, and redox biology. The article describes the critical role of metabolic and redox processes in aging, with focus on mitochondrial function and metabolic pathways (*e.g.*, autophagy and mitophagy), suggesting that understanding these can lead to strategies for extending healthspan and lifespan. The paper discusses the “Mitochondrial dysfunction theory of aging” and the “Hormesis theory of aging,” proposing that while mitochondrial dysfunction can contribute to aging, there are beneficial aspects to mitochondrial stress that can extend lifespan. The paper also touches on the importance of diet and lifestyle changes in modulating aging processes, highlighting the potential of interventions like dietary restriction, exercise, and pharmacological agents that target key metabolic pathways to promote healthier aging. Additionally, the article delves into the evolving understanding of redox biology, particularly the dual roles of reactive oxygen species (ROS) as both damaging agents and signaling molecules. This nuanced view challenges the traditional “Free Radical Theory of Aging” and suggests a more complex relationship between redox states and aging. Overall, the paper advocates for a more integrated view of metabolism, redox biology, and aging, calling for continued multidisciplinary research to uncover the molecular mechanisms that link these processes to age-related changes and diseases.

Zhang and colleagues explore the modification of proteins by O-GlcNAcylation in the context of neurodegenerative diseases and aging. O-GlcNAcylation, a post-translational modification, plays a critical role in cellular processes by responding to nutrient availability and stress. This study particularly investigates how this modification may influence neurodegenerative disorders like Alzheimer’s disease (AD) and Parkinson’s disease (PD) through changes in the proteome of the mouse cortex. The authors employed mass spectrometry to identify proteins that undergo O-GlcNAcylation, finding significant alterations in proteins involved in synaptic function and trafficking—areas crucial to neurodegenerative disease mechanisms. They discovered that increasing O-GlcNAc levels through the inhibitor Thiamet G (TG) affects the phosphorylation of tau, a protein associated with AD, suggesting therapeutic potential. However, the study also notes that elevated O-GlcNAc levels can impair cognitive functions, highlighting a complex balance between beneficial and detrimental effects. Key findings include the identification of specific proteins such as DNAJC6 and PICALM, which are known risk factors for PD and AD, respectively. These proteins showed changes in their O-GlcNAcylation status, linking this modification to the pathology of these diseases. The study suggests that modulating O-GlcNAcylation could be a strategy for targeting molecular pathways implicated in aging and neurodegenerative diseases by linking metabolic state (through O-GlcNAcylation) to neurodegenerative changes.

Fernandez-Abascal and Artal-Sanz explore the role of prohibitins (PHB) in neurodegeneration and mitochondrial homeostasis, focusing on their significance in age-related neurodegenerative disorders. They discuss the increased incidence of such disorders with rising life expectancy and the challenge of diagnosing them at an advanced stage. Prohibitins, evolutionary conserved within the mitochondrial PHB complex, are highlighted for their regulatory functions in aging, metabolism, and association with neurodegenerative diseases, like Alzheimer’s, Parkinson’s, and amyotrophic lateral sclerosis through unclear mechanisms. The review consolidates current research on the involvement of PHB in synaptic functions and their protective *versus* toxic effects under different conditions. It emphasizes mitochondrial stress as a common starting point for neurodegeneration, proposing PHB and mitochondrial pathways as potential targets for early diagnosis and treatment.

Chatham and colleagues expertly explore the intricacies of calcium (Ca2+) signaling modulation by STIM (Stromal Interaction Molecule) and Orai proteins, emphasizing their significance in the physiology of aging and age-associated diseases. The tight regulation of intracellular Ca2+ concentrations is crucial for numerous cellular functions, including cell survival, metabolism, and transcription. STIM and Orai proteins, being highly conserved and central to mammalian Ca2+ signaling systems, play pivotal roles in Store-Operated Calcium Entry (SOCE), directly impacting cellular homeostasis. The structural and functional nuances of STIM1, STIM2, Orai1, Orai2, and Orai3 proteins are also outlined, illustrating how their interactions mediate calcium influx into cells. STIM and Orai dysregulation are implicated in various aging-related conditions, notably cardiovascular diseases and neurodegeneration. This process is particularly significant in the context of neurodegeneration, cardiovascular diseases, and cellular aging, where calcium dysregulation is a common theme. The authors emphasize the therapeutic potential of targeting these proteins to ameliorate age-related pathologies, highlighting the need for further research to fully understand their roles in aging and disease progression.

Zaburdaev and colleagues reported their mathematical modeling study that if ethanol is supplied periodically, with certain frequency and concentration, *C. elegans* dauer can live toward an unlimited lifespan. The authors recently have published studies that show that *C. elegans* dauer can use ethanol as an external energy source, and upregulate metabolic enzymes including SODH-1 and ALH-1, that catalyze the conversion from ethanol to acetaldehydrate and then to acetate. As mitochondrial deterioration was found to precede the death of worms, the authors make the assumption that if mitochondria can regenerate and detoxification can happen, then worms would have a longer lifespan. Then periodic ethanol with optimal feeding period was shown advantageous over constant ethanol in the modeled lifespan. Overall a provocative idea.

Chen and Dodson provided critical review and literature, and insights into Nrf2 in aging, metabolism and redox biology. Nrf2 regulates the expression of key genes involved in redox regulation, and there are much evidence that its loss of function significantly exacerbates many disease phenotypes. This article highlighted its involvement in multiple neurodegenerative diseases. Furthermore, the authors reviewed approaches with gene delivery, antisense oligos, pharmacological compounds, and those targeting upstream co-activators and downstream effector have been tested to target Nrf2 with the aim to protect against age related pathologies. As accumulation of cellular damage has been shown a critical hallmark of aging, further understand the role of Nrf2 and its regulation is essential for developing a better approach to promote healthy aging.

Gupta and colleagues reviewed the interaction of BAG5 with heat shock proteins, and PINK1, by which BAG5 plays a role in protein quality control, with special emphasis on cardiovascular and neurodegenerative diseases. As BAG5 is one of the key plays in the junction of autophagy, mitophagy, ER-mediated pathways, understanding the molecular mechanisms of how BAG5 interact with other cellular constituents provide a foundation for tackling this regulatory node for therapeutic interventions of age-related chronic diseases.

McKenna and Tong and colleagues reported in a research article that PU.1/Spi1 knockout in the adipocytes resulted in higher energy expenditure in males at 4–5 months of age, and higher insulin sensitivity and lower adiposity at 10–11 months of age. RNAseq analyses showed PU.1 regulated inflammatory and thermogenic programs. This research represents one of the first steps to fully appreciate the role of PU.1 in different cells and tissues as a contributor of metabolic regulation which is essential for health in the process of aging.

Finally, Borras provided considerable thoughts and significant insights on the challenges of unlocking the biological secretes of aging, including the process, the theories, the models and the biomarkers. There is so much that we do not know. First of all, fundamentally what is aging? There is a nearly continuous process of changes in nearly all cells, tissues, and organisms, with time passing by. However, at what point are these changes deleterious? Whether/when/what early changes pre-destine late changes? What regulates this aging process? Is it really irreversible? Associated with unknowns, there are a lot of theories of aging proposed over the years. And there are *in vivo* models investigators use to identify mechanisms and interventions. Dr. Borras provided summary of the theories and the models with strengths and limitations, which is a great service to the field and helpful to trainees in the aging field to gain an overview. Intertwined with the process and the theories of aging are the hallmarks and biomarkers which often reflect but one aspect of the aging processes and are dependent on models and interventions. Because of these challenges, the search remains on and intense in the pursuit to unlock the secrets of aging.

We hope that this Research Topic provides new insights in aging research, in particular metabolism, redox regulation, protein quality control, and current development and challenges.

